# Kinase CDK2 in Mammalian Meiotic Prophase I: Screening for Hetero- and Homomorphic Sex Chromosomes

**DOI:** 10.3390/ijms22041969

**Published:** 2021-02-17

**Authors:** Sergey Matveevsky, Tsenka Chassovnikarova, Tatiana Grishaeva, Maret Atsaeva, Vasilii Malygin, Irina Bakloushinskaya, Oxana Kolomiets

**Affiliations:** 1 Laboratory of Cytogenetics, Vavilov Institute of General Genetics, Russian Academy of Sciences, 119991 Moscow, Russia; grishaeva@vigg.ru (T.G.); olkolomiets@mail.ru (O.K.); 2 Department of Animal Diversity and Resources, Institute of Biodiversity and Ecosystem Research, Bulgarian Academy of Science, 1000 Sofia, Bulgaria; t.tchasovnikarova@gmail.com; 3 Department of Zoology, Biological Faculty, University “Paisi Hilendarski”, 4000 Plovdiv, Bulgaria; 4 Department of Cell Biology, Morphology and Microbiology, Chehen State University, 364051 Grozny, Russia; acaeva-mm@mail.ru; 5 Department of Vertebrate Zoology, Biological Faculty, Lomonosov Moscow State University, 119991 Moscow, Russia; vmalygin@mail.ru; 6 Laboratory of Genome Evolution and Speciation, Koltzov Institute of Developmental Biology, Russian Academy of Sciences, 119334 Moscow, Russia; irina.bakl@gmail.com

**Keywords:** cyclin-dependent kinase 2, CDK2, SUN1, MLH1, *Rattus*, *Ellobius*, *Nannospalax*, *Microtus*, *Cricetulus*, meiosis, synaptonemal complexes, sex chromosomes

## Abstract

Cyclin-dependent kinases (CDKs) are crucial regulators of the eukaryotic cell cycle. The critical role of CDK2 in the progression of meiosis was demonstrated in a single mammalian species, the mouse. We used immunocytochemistry to study the localization of CDK2 during meiosis in seven rodent species that possess hetero- and homomorphic male sex chromosomes. To compare the distribution of CDK2 in XY and XX male sex chromosomes, we performed multi-round immunostaining of a number of marker proteins in meiotic chromosomes of the rat and subterranean mole voles. Antibodies to the following proteins were used: RAD51, a member of the double-stranded DNA break repair machinery; MLH1, a component of the DNA mismatch repair system; and SUN1, which is involved in the connection between the meiotic telomeres and nuclear envelope, alongside the synaptic protein SYCP3 and kinetochore marker CREST. Using an enhanced protocol, we were able to assess the distribution of as many as four separate proteins in the same meiotic cell. We showed that during prophase I, CDK2 localizes to telomeric and interstitial regions of autosomes in all species investigated (rat, vole, hamster, subterranean mole voles, and mole rats). In sex bivalents following synaptic specificity, the CDK2 signals were distributed in three different modes. In the XY bivalent in the rat and mole rat, we detected numerous CDK2 signals in asynaptic regions and a single CDK2 focus on synaptic segments, similar to the mouse sex chromosomes. In the mole voles, which have unique XX sex chromosomes in males, CDK2 signals were nevertheless distributed similarly to the rat XY sex chromosomes. In the vole, sex chromosomes did not synapse, but demonstrated CDK2 signals of varying intensity, similar to the rat X and Y chromosomes. In female mole voles, the XX bivalent had CDK2 pattern similar to autosomes of all species. In the hamster, CDK2 signals were revealed in telomeric regions in the short synaptic segment of the sex bivalent. We found that CDK2 signals colocalize with SUN1 and MLH1 signals in meiotic chromosomes in rats and mole voles, similar to the mouse. The difference in CDK2 manifestation at the prophase I sex chromosomes can be considered an example of the rapid chromosome evolution in mammals.

## 1. Introduction

Evolutionary biologists use a wide range of methods and approaches to characterize various phenomena. The evaluation of chromosomal evolutionary features can be carried out both using classical G- and C-banding, and the identification of genetic and protein markers in the chromosome sets. Chromosome-specific probes, clones of bacterial artificial chromosome (BAC), and conserved protein complexes can serve as such markers. Cyclin-dependent kinase 2 can be used as a functional marker for these purposes.

Cyclin-dependent kinases (CDKs) are heteromeric serine/threonine kinases that participate in cell cycle regulation in association with activating substrates, termed cyclins [1]. To date, over 20 CDK genes are known [2,3], several of which drive the cell cycle [4]. Proliferating cells pass G1, progress with DNA replication through S phase, and then after the gap phase (G2), enter a single mitotic division, producing genetically identical cells. Unlike mitosis, in meiosis, the extended prophase is followed by two specific meiotic divisions, featuring pairing and recombination between homologous chromosomes during the first reductive meiotic cell division. In contrast, the second meiotic division is an equational division, resulting in the production of genetically different haploid cells. Different CDKs regulate the G1/S-phase transition and DNA replication in mitotic cell divisions, the cyclin E/CDK2 complex is a crucial driver in the transition from G1 to S-phase, and the cyclin A/CDK2 complex is necessary for S-phase progression [5]. CDK2 and CDK1 participate in the DNA double-strand breaks (DSBs) repairing during mitosis [6], but only CDK2 is essential for meiosis [7]. While CDK2 is listed as one of many proteins involved in meiosis [8], its role is significant. Both male and female *Cdk2*-mutant mice suffer from sterility due to germ cell death in meiotic prophase I [9,10]. Moreover, loss of CDK2 catalytic function inevitably leads to sterility due to a block in the progression of meiosis I [11]. In mutant *Cdk2*^-/-^ mice, localization of DSB markers (RPA and RAD51) in zygotene and pachytene spermatocytes was close to normal; however, the MLH1 protein was absent at the pachytene stage [10]. These data clarify that DSB repair begins without requiring CDK2 but cannot be completed without it. The crucial input of CDK2 for meiotic crossover formation was shown recently by Palmer et al. [12]. To date CDK2 is considered to play an essential role in chromosome synapsis, meiotic recombination, and formation of the sex body (special chromatin-modified domain of the male sex chromosomes at the periphery of the nucleus) during the first meiotic prophase. Furthermore, synthesis and degradation of CDK2 are crucial for the progression of meiosis [10,13,14].

Most eutherian mammals, including rodents, possess a sex chromosome system in which the male pair is heteromorphic (XY), and the female pair is homomorphic (XX). As Kauppi et al. [15] have stated, sex chromosomes are the “Achilles’ heel” of meiosis in male mammals. This is primarily due to the evolutionary consequences of male sex chromosome degradation, where the two sex chromosomes, X and Y, retain only a short homologous region, the so-called pseudoautosomal region (PAR) [16]. The non-homologous asynaptic (unpaired) axes are forced to undergo meiotic sex chromosome inactivation (MSCI) for correct passing of checkpoints in meiotic prophase I. Otherwise, cells may be arrested. It is known that, in autosomes, the DSB repair is already completed at the pachytene stage, and in the X chromosome, at the diplotene stage [17]. In males, the number of DSBs in the X and Y chromosomes differs, always being higher in the X chromosome [18]. In the Y chromosome, mandatory single DSBs are formed in the PAR at the leptotene stage.

The study of CDK2 manifestation and function in meiotic progression has been conducted only in laboratory mice [9,10,12,19]. In this case, the analysis of such a multifunctional molecule as CDK2 on other animals can be promising. Evolution has led to a diversity of sex chromosomes among the distinct phyla of animals, and rodents in particular (for example [20,21]. This study aimed to confirm the specifity of a kinase (CDK2) meiotic manifestation in the sex chromosomes of various evolutionary stories. Analysis of the behavior of the meiotic chromosomes in prophase I was carried out using the immunocytochemical study of synaptonemal complexes (SC), the multi-protein structures that facilitate synapsis of homologous chromosomes [22]. In addition to CDK2 antibodies to identify this kinase, we used antibodies to proteins RAD51 and MLH1 (a mismatch repair protein) as markers for DNA double-strand breaks and recombination [23,24]. We studied seven rodent species from four subfamilies and three families with various sex chromosome systems, reflecting different evolutionary stages (Table S1, Figure S1): The Norwegian rat *Rattus norvegicus* (Muridae), with XX-XY and PAR, the common vole *Microtus arvalis* with XX, achiasmatic XY and no PAR; the lesser mole rat *Nannospalax leucodon* (Spalacidae), with XX-XY and PAR; the gray dwarf hamster *Cricetulus migratorius* (Cricetidae), with XX-XY, PAR and equal-length heteromorphic chromosomes; and three species with isomorphic (homomorphic) sex chromosomes for both sexes: The northern mole vole *Ellobius talpinus*, the eastern mole vole *E. tancrei*, and the Alay mole vole *Ellobius alaicus* (male XX with broad central asynaptic zone [25,26] and female XX with delayed synapsis [27]). The primary focus was on the classical system XX-XY (rat) and the deviant XX-XX (the northern mole voles).

## 2. Results

The major protein of SC, SYCP3, indicates the structure and behavior of axial/lateral elements in prophase I [28] and CREST marks the proteins of kinetochores [29]. We used single-, double-, and triple-round immunostaining to analyze the distribution of these proteins and kinase CDK2 in meiotic nuclei (Figures S2, S3 and combination 1 in Figure S4).

**Figure 1:**
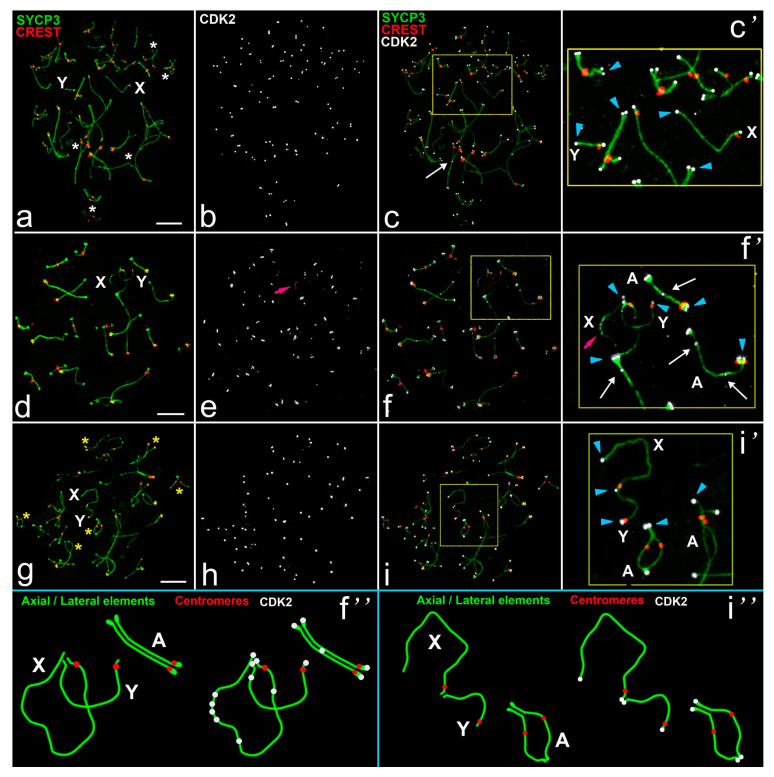
CDK2 localization in meiotic chromosomes of the rat *R. norvegicus*. SCs were immunostained using antibodies to SYCP3 protein (green), centromeres using antibodies to proteins of kinetochores (CREST, red), and CDK2 kinase using antibodies to CDK2 (white). Blue arrowheads point to the telomeric localization of Cdk2. White arrows point to the interstitial CDK2 location. Pink arrows outline numerous CDK2 signals in asynaptic segments of the sex bivalent. A—autosomal bivalents. XY—sex chromosomes. (**a**–**c**) Mid zygotene. Asynapsis in several bivalents is marked as a white stars; (**d**–**f**) Mid pachytene; (**g**–**i**) Diplotene. Desynapsis in several bivalents is marked as yellow stars. (**c’**,**f’**,**i’**)—enlarged parts of the relevant photos. (**f’**,**i’**)—schemes of some meiotic chromosomes in (**f’**,**i’**). Scale bar (**a**–**i**) = 5 µm.

**Figure 2:**
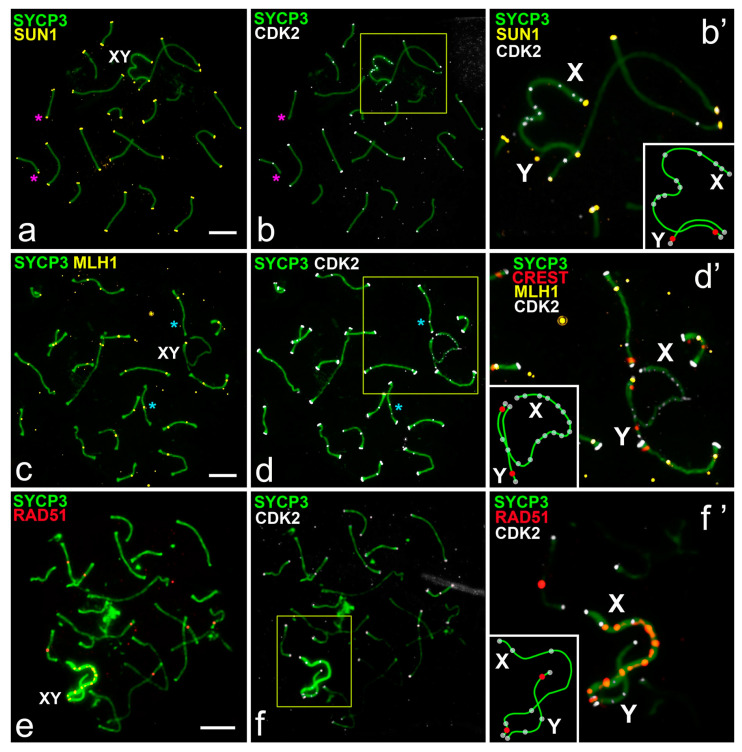
CDK2, SUN1, MLH1, and RAD51 distribution in the pachytene spermatocytes of the rat *R. norvegicus*. SCs were immunostained using antibodies to SYCP3 protein (green), and centromeres using antibodies to proteins of kinetochores (CREST, red). Schemes of sex chromosome synapsis, centromeric regions (red) and CDK2 foci (white) are shown in the insets (**b’**,**d’**,**f’**). Pink stars pointed to non-colocalization of the SUN1 and CDK2 foci in some autosomal bivalents. Blue stars pointed to non-colocalization of the MLH1 and CDK2 foci in some autosomal bivalents. (**a**,**b**) CDK2 (white) and SUN1 (yellow) colocalized at the telomeric sites of SC. CDK2 signals were detected in asynapsed parts of the X chromosome (**b’**). (**c**,**d**) Colocalization of the CDK2 (white) and MLH1 (yellow) at the interstitial sites of autosomal SCs. The X chromosome showed numerous CDK2 foci, whereas the Y showed single CDK2 foci. Both sex chromosomes had CDK2 foci in the telomeric parts (**d’**). (**e**,**f**) CDK2 (white) and RAD51 (red) signals did not colocalize and had different positions in chromosomes. There were numerous RAD51 foci on the X chromosome, whereas the Y lacks these signals. Rare signals of CDK2 were detected on the Y chromosome (**f’**). Scale bar (**a**–**f**) = 5 µm.

In rats (Figure 1a–i and Figure 2a–f), during the zygotene stage, axial elements initiated telomeric or interstitial synapsis in autosomes (Figure 1a). Sex chromosomes were detected as univalents (Figure 1a,c’). In the mid zygotene, CDK2 foci were localized in the telomeric sites of the axial elements (Figure 1a–c’). In only a few partially synapsed SC bivalents, CDK2 was localized to interstitial sites. In the mid pachytene, 20 autosomal bivalents and a sex bivalent were formed. CDK2 localized to both telomeric sites and interstitial sites along SCs (Figure 1d–f’ and Figure 2b,d,f). A short (Figure 1d,f’) or longer (Figure 2a–d) synaptic region between X and Y chromosomes was detected. For the first time, we detected that centromeric regions of sex chromosomes were not co-oriented: A centromere in X was inside the synaptic site, but in the Y the centromere was at the pretelomeric end of the asynaptic axis. This important and unusual feature of male meiosis in rats was previously unknown. In the sex bivalent, a strong CDK2 signal appeared in the telomeric sites, and weaker dot-like foci were seen along asynaptic parts of the X (15.8 ± 0.6 foci) and Y (1.42 ± 0.17) (Figure 1f–f″ and Figure 2b’,d’,f’, Figure S5). In the diplotene, CDK2 foci were detected in the telomeric sites of the desynapsed autosomal bivalents only (Figure 1g–i″).

**Figure 3:**
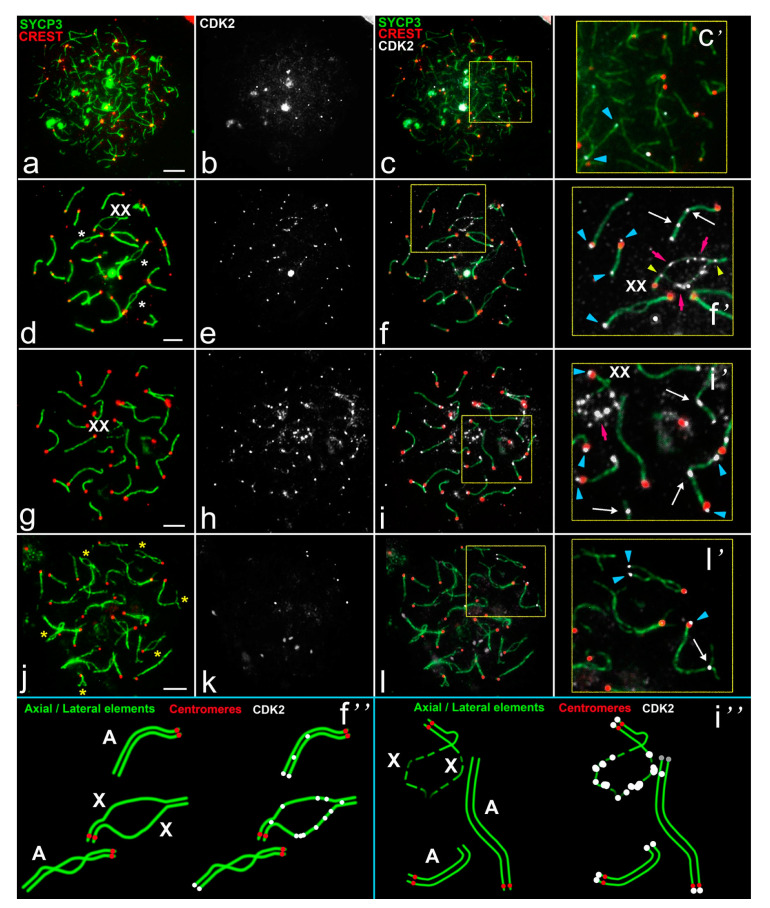
Cdk2 localization in meiotic chromosomes of spermatocytes of the mole vole *E. talpinus*. SCs were immunostained using antibodies to SYCP3 protein (green), centromeres using antibodies to proteins of kinetochores (CREST, red), and kinase CDK2 using antibodies to CDK2 (white). Blue arrowheads point to the telomeric CDK2 localization. White arrows pointed to the interstitial CDK2 localization. Pink arrows outline numerous CDK2 signals in asynaptic segments of the sex bivalent. (**a**–**c**) Late leptotene—early zygotene. (**d**–**f**) Late zygotene—early pachytene. White stars mark asynaptic regions in the bivalents. Yellow arrowheads point to two CDK2 signals in the synaptic segments in the sex bivalent. (**g**–**i**) Mid pachytene. (**j**–**l**) Diplotene. Desynapsis in several bivalents was marked as yellow stars. (**c’**,**f’**,**i’**,**l’**)—enlarged parts of the relevant photos. (**f’**,**i’**)—schemes of meiotic chromosomes in (**f’**,**i’**). Scale bar (**a**–**l**) = 5 µm.

**Figure 4:**
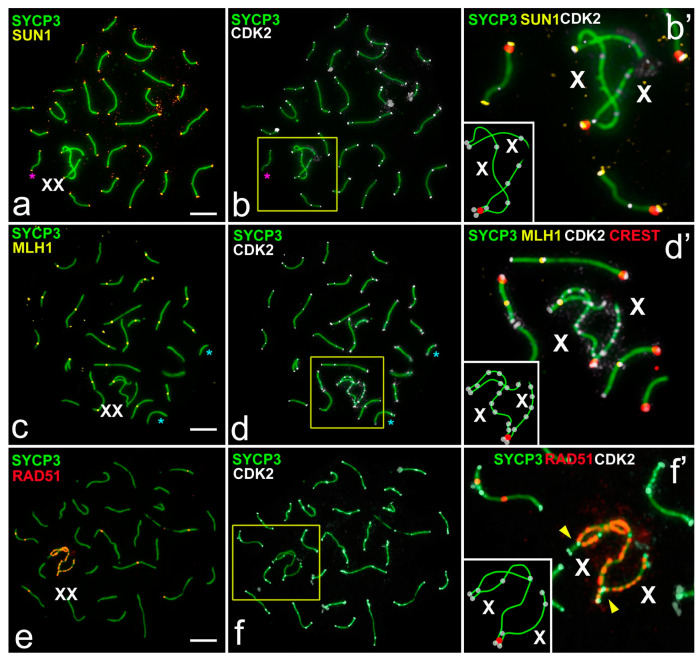
CDK2, SUN1, MLH1, and RAD51 distribution in meiotic chromosomes of spermatocytes of the mole vole *E. talpinus*. SCs were immunostained using antibodies to SYCP3 protein (green), centromeres–antibodies to proteins of kinetochores (CREST, red), and kinase CDK2 using antibodies to CDK2 (white). Schemes of sex chromosome synapsis, centromeric regions (red), and CDK2 (white) foci are shown in the insets (**b’**,**d’**,**f’**). Pink stars point to non-colocalization of the SUN1 and CDK2 foci in autosomal bivalent. Blue stars point to non-colocalization of the MLH1 and CDK2 foci in some autosomal bivalents. (**a**,**b**) CDK2 (white) and SUN1 (yellow) colocalized in the telomeric sites of chromosomes. CDK2 signal was detected in asynaptic parts of the X chromosomes (**b’**); (**c**,**d**) Colocalization of CDK2 (white) and MLH1 (yellow) in the interstitial sites of autosomal SCs. Asynaptic parts of the sex (XX) bivalent demonstrated numerous CDK2 foci, clear CDK2 signals in the telomeric sites, and one CDK2 focus in one of the synaptic segments (**d**,**d’**); (**e**,**f**) CDK2 (white) and RAD51 (red) signals did not colocalize and had different positions in the chromosomes. Asynaptic parts and one of the synapsed segments showed numerous RAD51 foci. CDK2 signals localized in the telomeric sites, at the MLH1-positive positions (yellow arrowheads, see (**c**) [25]), and in the asynaptic parts of the XX bivalent (**f’**). Scale bar (**a**–**f**) = 5 µm.

In the mole voles *E. talpinus* (Figure 3a–l and Figure 4a–f), SYCP3 was visualized from the early leptotene to the diplotene/diakinesis and SYCP3 dynamics is similar to the described one in other rodents. In the late leptotene to early zygotene, this protein began to form separate axial elements, which entered synapsis at the mid-late zygotene stages (Figure 3a,d). Chromosome synapsis completed in the mid pachytene, when fully formed SCs became visible (Figure 3g). In the diplotene, chromosomes underwent desynapsis (Figure 3j). The immunodetection of the centromeres proved that, in the northern mole vole, all bivalents (26 autosomal and a sex bivalent) were formed by acrocentric chromosomes (Figure 3a,d,g,j).

CDK2 foci localized at the ends of the single axes of autosomal bivalents only (Figure 3b–c’). In the late zygotene to early pachytene axial elements began to synapse, and some of the autosomal bivalents retained unsynapsed parts (Figure 3d). Sex bivalents (XX) had pretelomeric synaptic segments and wide unsynapsed areas between them (Figure 3d), which was specific to the males of the species [25,30]. The nucleolus-like body or chromatin dense body (ChB) was formed within one of the axial elements of the asynaptic zone. The ChB was SYCP3 negative, so we see an SYCP3-axis interruption [25,30].

Inside the sex bivalent, numerous CDK2 foci localized along asynapsed segments; there were single CDK2 signals in one or both synaptic segments (Figure 3e–f’ and Figure 4b,d,f). In the mid pachytene, all bivalents fully synapsed except the sex bivalent. At this stage, numerous CDK2 signals were detected in the asynaptic zone of the sex bivalent (9.83 ± 0.49 foci for first X and 8.01 ± 0.39 for second X) (Figure 3g–i’ and Figure S5). Autosomal bivalents demonstrated telomeric and interstitial localizations for CDK2 on the fully synapsed chromosomes (Figure 3f,f’,f″,i,i’,i″). Progressive desynapsis of homologs occurred during the diplotene (Figure 3j), and only a few bivalents retained telomeric and interstitial CDK2 sites (Figure 3k–l’).

The SUN1 protein participates in telomere attachment to the nuclear envelope and correct synapsis of chromosomes in the prophase of meiosis [31,32]. We used SUN1 antibodies as a marker for telomeres. In the pachytene, the chromosomes of rats and mole voles showed SUN1 foci, which were clearly localized in the telomeric regions.

Simultaneous immunostaining of the SUN1 and CDK2 (combination 2, Figure S4) allowed us to confirm their colocalization in the telomeres of all autosomal SCs and sex bivalents (Figure 2a–b’ and Figure 4a–b’). In some bivalents one telomere site was SUN1- and CDK2-positive simultaneously, while the other was either SUN1- or CDK2-positive or did not show these signals at all. Only 12 of 420 (rat) and 7 of 540 (mole vole) autosomal and XY bivalents did not have colocalization of SUN1 and CDK2 signals (number of cells = 20 for both species) (Figure 2a,b and Figure 4a,b, Table S3).

The most interesting question was the investigation of the colocalization of MLH1 and CDK2 in sex chromosomes. MLH1 protein was used as a marker for recombination sites [33]. We performed sequential immunostaining for the assessment of joint localization of MLH1 and CDK2 (combinations 3 and 4, Figure S4). One or two MLH1 foci were detected in interstitial parts of rat and mole vole autosomes during pachytene (Figure 2c and Figure 4c). CDK2 foci were located at the same sites in the vast majority of autosomal bivalents (Figure 2c–d’ and Figure 4c–d’). However, in some autosomal bivalents, the MLH1 and CDK2 signals were not colocalized (non-colocalization) (Figure 2c,d). Our calculations established that the level of non-colocalization is about 5.5% (1.1 ± 0.23 bivalents per nucleus) for rats and 2.3% (0.6 ± 0.15) for mole voles (Table S4). In the synaptic parts of the mole vole sex bivalent, single MLH1 foci were distinguished (Figure 4d’). It was shown that location of the MLH1 signals in the XX bivalent might be variable; more often, a single signal was detected in one of the synaptic segments [25]. In sex bivalents, CDK2 foci were visualized at both telomeric and MLH1 sites, and in asynapsed regions (Figure 2d’ and Figure 4d’,f’) in both mole voles and in rats. It is worth noting that in the sex bivalent of rats, the X chromosome displayed significantly more CDK2 foci compared to the Y chromosome (Figure 2b’,d’).

We next investigated an interaction between a DSB repair marker, RAD51 [34], and CDK2 foci. We observed localization of the signals of RAD51 and CDK2 using combination 5 and 6 (Figure S4). In rat and mole vole spermatocytes, RAD51 protein was distributed irregularly along autosomes: In the mid pachytene the number of RAD51 foci varied from zero to three (Figure 2e and Figure 4e). In the sex bivalent of rats, RAD51 was detected in the X chromosome, whereas in Y, if there were signals at all, they were rare and weak (Figure 2f’). In the XX bivalent of mole voles, numerous RAD51 foci were present along the asynaptic axes (Figure 4e,f’). Several RAD51 signals were detected in synapsed segments of male XX bivalents (Figure 4f’). CDK2 signals never exactly matched with RAD51 signals along asynaptic axes, although single signals could partially overlap in some cases (Figure 2e,f’ and Figure 4e,f’).

**Figure 5:**
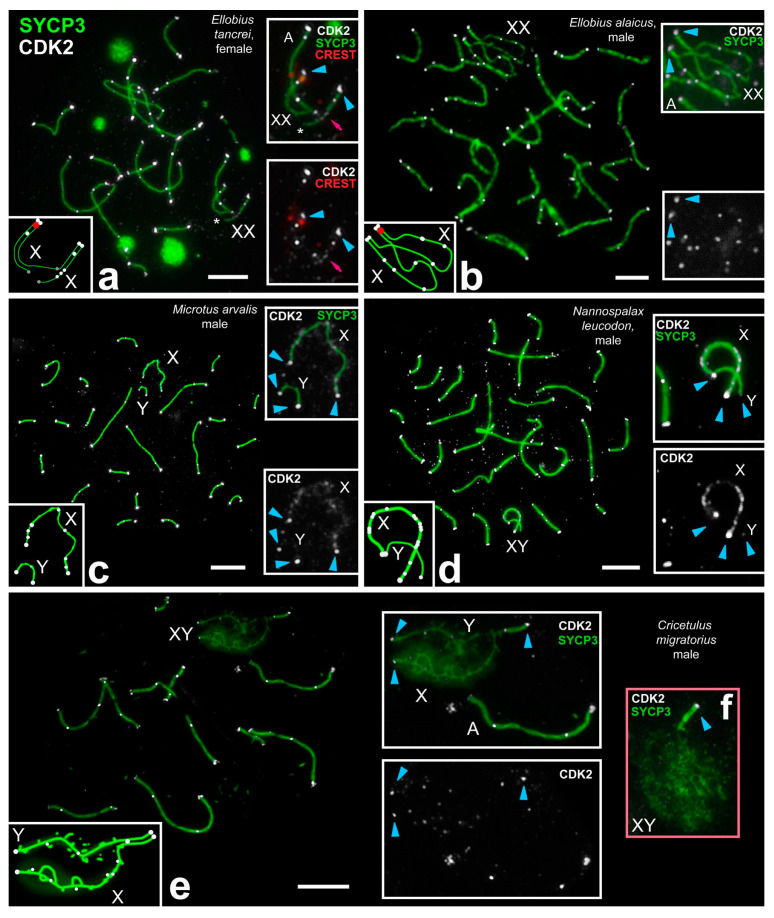
CDK2 localization in meiotic chromosomes of males and females of distinct rodent species. In all species, CDK2 (white) localized in the telomeric and interstitial sites of autosomes (A). Blue arrowheads point to telomeric CDK2 localization in sex bivalents (XY). Schemes of sex chromosome synapsis, centromeric regions, and CDK2 dots are shown in the left bottom insets (**a**–**e**). (**a**) Pachytene oocyte of the mole vole *E. tancrei*. An incomplete synapsis occurred in the central part of the sex (XX) bivalent (white star). There were different CDK2 signals: Strong ones in the telomeric and interstitial sites, and weak signals in the synapsed parts of the sex bivalent (pink arrow). (**b**) Pachytene spermatocytes of the mole vole *E. alaicus*. CDK2 signals were detected in the telomeric and interstitial sites for all bivalents. A weak CDK2-cloud was visualized around the asynaptic area of XX. (**c**) In the pachytene spermatocyte of the vole *M. arvalis* X and Y were asynaptic (without synapsis between them). The X chromosome showed numerous CDK2 foci (not intense here). The Y chromosome showed CDK2 foci in telomeric sites only. (**d**) In the pachytene spermatocyte of the mole rat *N. leucodon* numerous CDK signals appeared in the asynaptic part of the X chromosome. Y had telomeric CDK2 foci only. (**e**) Pachytene spermatocytes of the hamster *C. migratorius.* Autosomes had the same regularities: Telomeric and one or two interstitial sites were CDK2-positive. Rare CDK2-dots were identified in asynaptic regions of XY. (**f**) In the late pachytene hamster spermatocyte, asynaptic X- and Y-axes had diffused SYCP3. Scale bar (**a**–**e**) = 5 µm.

A similar but not identical CDK2 distribution in the pachytene sex chromosomes was revealed for some rodents (Figure 5a–e). The distribution of CDK2 signals in males of *E. alaicus* was similar to *E. talpinus* though fewer foci were observed in the asynapsed regions of *E. alaicus* (Figure 5b, Figures S1c and S5). Although the differences between the two X chromosomes of the Aalay mole vole were not significant (**X**: 7.87 ± 0.67 foci; X*: 9.84 ± 0.63 foci; Table S2, Figure S5). Differences between species were significant (Table S2). The female sex (XX) bivalent of *E. tancrei* retained asynapsis in the central region (delayed synapsis) and had weak CDK2 signals in synaptic and asynaptic regions (3.63 ± 0.36 for XX bivalent) and strong CDK2 signals at telomeric and interstitial sites (Figure 5a, Figures S1d, S5, Table S2). CDK2 distribution in the female XX was similar to autosomal bivalents.

The assessment of CDK2 distribution in each sex chromosome of *N. leucodon* was carried out at the early to mid-pachytene stage transition, since in the mid pachytene Y completely synapses with X [35]. Intense CDK2 signals were observed both in the telomeres of the sex chromosomes and along the entire asynaptic X axis (10.37 ± 0.74 foci) (Figure S5). At the same time, Y either does not have CDK2 foci, or they were extremely faint (1.2 ± 0.16) (Figure 5d, Figures S1g, S5, Table S2).

The sex chromosomes of *C. migratorius* had CDK2-positive telomeres and one strong CDK2 focus on the synaptic segment (Figure 5e, Figure S1e). Asynaptic X- and Y-axes had diffused SYCP3 and rare separate CDK2 dots in *C. migratorius* (**X**: 5.76 ± 0.44 foci; **Y**: 4.47 ± 0.43 foci) (Figure 5e,f, Figures S1e, S5, Table S2).

The asynaptic X and Y chromosomes of *M. arvalis* had intense CDK2 foci in telomeric regions at different prophase I stages (Figure 5c, Figures S1a and S3). Faint diffuse or dot-like multiple CDK2 signals were observed along X (5.4 ± 0.48 foci) at mid pachytene, while along Y chromosome one weaker dot-like signal, or no signal, was present (1.06 ± 0.13 signal) (Figure 5c, Figures S1a, S3a–c, S5, Table S2). In the second half of prophase I, CDK2 signals were retained only in the telomeres of chromosomes, including the sex bivalent (Figure S3d–e), and when SYCP3 degrades at diakinesis, CDK2 foci were absent (Figure S3f).

An essential indicator of kinase pattern is the ratio of the average number of CDK2 signals to the average length of sex chromosomes (Table S2). Short Y chromosomes (rat, mole rat, vole, and hamsters) had similar CDK2 values (0.22–0.3). Long X chromosomes of the rat, mole rat, and both male mole voles had similar values (0.8–1.3), with the exception of vole, hamster, and mole vole female.

## 3. Discussion

Implementing the multi-round immunocytochemical technique allowed us to discover the pattern of CDK2 distribution and its colocalization with other proteins (or its absence) in prophase I of meiosis in seven animal species. Ashley et al. established the colocalization of CDK2 and MLH1 [19], an unpaired base repair protein (mismatch repair protein), and a marker of late recombination nodules. Although several autosomal bivalents did not show joint MLH1 and CDK2 localization, the level of their colocalization was high, approximately 94.5% (rat) and 97.7% (mole vole) of the total number of autosomes studied (Table S4). It is worth noting that in [19], one of the mouse nuclei also contained bivalents, where, in the interstitial regions, the CDK2 signal was absent in the MLH1-positive dots or the MLH1 focus was absent in the CDK2-positive dots. No explanation has been given for this phenomenon; however, one can hypothesize that specifics in synapsis progression of individual autosomes, delay or advancement occur. It is believed that CDK2 interacts with HEI10 and RNF212 ligases and they are pro-crossover factors involved in MLH1 and MLH3 recruiting and, accordingly, maturation of late recombination nodules [36]. Here, we demonstrated the colocalization of CDK2 and MLH1 at recombination sites of sex chromosomes in those species for which this synapsis occurs in short segments of heteromorphic chromosomes, as well as in species with isomorphic sex chromosomes. In addition, we revealed CDK2 signals of lower intensity along the axes of the prophase I achiasmatic sex chromosomes, similar to other species. These findings pose the question of whether there is some unknown role for CDK2 in sex chromosomes function.

In the rat meiotic prophase chromosomes, CDK2 and MLH1 were colocalized, with one clear CDK2 signal present in each synaptic segment of the sex bivalent, in which the MLH1 signal was immunodetected. It is evident that in the studied rodents, CDK2 kinase participates in the formation of recombination nodules, as previously seen in mouse experiments.

In the asynaptic region of the Y, RAD51 signals are single or absent, as shown in mice [37] and here in male rats. DSB repair in asynaptic regions of sex chromosomes (the regions lacking the homologous repair pathway) is likely dependent on intact sister chromatids, as is the case in autosomes [38]. On the other hand, in meiotic prophase I, DSBs repair in unpaired X- and Y-axes is done under MSCI proteins. This explains the prolongation of the repair until the diplotene stage [17,39].

Unlike autosomes and X, the Y is the only chromosome in most mammalian karyotypes that does not have a full homologous pair and never synapses entirely. In that respect, it is impossible to exclude either partial loss of sites in the Y, where DSBs can occur, or special chromatin modification (reorganization) in the same Y chromosome regions.

Our data on RAD51 and CDK2 distribution in the sex bivalents in males and females for two mole vole species (*E tancrei* and *E. talpinus)* with identical (isomorphic) X chromosomes are of great interest. In females, the XX chromosomes synapse completely [30], but often with delayed synapsis (Figure 1). In males, XX chromosomes form a closed sex bivalent with large areas of asynapsis in which chromatin is inactivated [25,30,40]. Although we did not observe identical CDK2- and RAD51-patterns, these signals could be located very close or even partially overlap. Both X chromosomes of the mole vole male had the ability to form numerous DSBs, unlike single DSBs in the synaptic part of the Y of the rat or mouse. Therefore, the extensive asynapsis between the male XX chromosomes is caused either by the absence of homology between them or, more likely, by a special epigenetic modification of chromatin. These findings suggest some input of CDK2 to the events of recombination.

Since the CDK2 immunolocalization in meiotic telomeres was established by Ashley et al. [19], some evidence of the important role of this kinase in attaching telomeres to the nuclear envelope has been revealed. It is known that the Sun-KASH multi-protein complex is responsible for the regulated motion of the meiotic chromosomes in the nucleus, which provides a binding site in the “nucleoskeleton–nuclear envelope–cytoskeleton” system [41,42,43]. One of the components of this complex is the SUN1 protein, which is involved in the attachment of meiotic telomeres to the inner membrane (INM) of the nuclear envelope [31,44]. Recent studies indicate that CDK2 is located in attachment plates of axial/lateral elements, where SUN1 is involved in telomere anchoring to the nuclear envelope [32]. In the *Cdk2*^-/-^ mutant mice, abnormal SUN1 localization in telomeres was observed, and it was concluded that CDK2 plays an important role in the correct distribution of SUN1 along the nuclear envelope [32]. The input of Speedy A, a noncanonical activator of CDKs, is also essential for telomere attachment to the nuclear envelope in mice [45]. We demonstrated that CDK2 and SUN1 were colocalized in meiotic telomeres in the spermatocytes of the rat and mole vole, which is similar to patterns described previously in mice [46,47]. The absence of colocalization in several bivalents is possibly associated with insufficient immunostaining of some segments of meiotic chromosomes.

In *N. leucodon,* CDK2 pattern is similar to that of a mouse, rat, and even vole. It is known that even within the same species, rapid rearrangements in sex chromosomes can occur, which are able to change protein patterns, such as MLH1 in another mole rat species, *N. ehrenbergi* [48]. Although our data [48] support assumption that the evolution of synapsis and recombination leads to the rapid evolution of sex chromosomes in *Nannospalax* group, we note a conservative kinase pattern for the lesser mole rat. 

Asynaptic sex chromosomes were described for several vole species and independent loss of the XY pairing was suggested [49]. The appearance of CDK2 telomeric signals on both sex chromosomes can be explained by a regulatory function of CDK2 in meiotic telomere attachment [47]. Nevertheless, the faint signals on the X are probably the product of dysregulated kinase activity in the case of newly asynapsed sex chromosomes.

**Figure 6:**
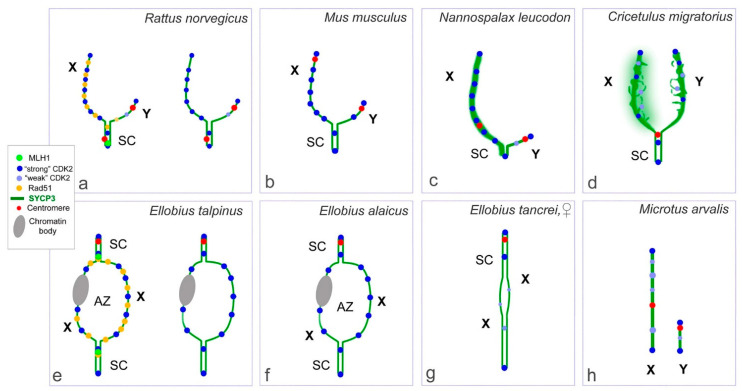
Schemes of CDK2 localization in pachytene sex chromosomes of the eight species of rodents. CDK2, RAD51, and MLH1 distributions are shown for males of *Rattus norvegicus* (**a**) and *Ellobius talpinus* (**e**). CDK2 distributions is shown for males of *Mus musculus* (**b**)*;* drawn based on previous studies [19,50,51,52]), *Nannospalax leucodon* (**c**), *Cricetulus migratorius* (**d**), *Ellobius alaicus* (**f**), *Microtus arvalis* (**h**), and females of *Ellobius tancrei* (**g**). In rat and mole voles sex bivalents, colocalization of MLH1 and CDK2 signals and close localization of RAD51 and CDK2 foci in synaptic segments, and different patterns of RAD51 and CDK2 foci in asynaptic parts are shown (**a**,**e**).

Here, we have analyzed five variants of rodent sex chromosome prophase I interactions and determined that the CDK2 signals were distributed in three different modes (Figure 6, Table S1):

- At the unsynapsed regions, the X chromosome has a lot of CDK2 signals, Y chromosome has no or one weak CDK2 focus:
  1. Classical synaptic XY:
    - PARs undergo synapsis, the centromeric region of the Y chromosomes is not involved in synapsis, as in *R. norvegicus* (compared with the mouse, in which the centromeric regions of both sex chromosomes are not involved in synapsis),
    - Synapsis between sex chromosome starts in their telomeric regions and then Y is synapsed to its full length with co-orientated centromeres, as in *N. leucodon*,
  2. Fully asynaptic X and Y, as in *M. arvalis*.
  3. Exclusive male XX, as in *E. talpinus* and *E. alaicus*, partial two-site synapsis.
- At the unsynapsed regions, the X and Y chromosomes have an average number of CDK2 signals, including SYCP3 loops and fragments:
  4. Synaptic XY: Sex chromosomes have the same or similar length with a long synaptic site and diffuse unsynapsed regions, as in *C. migratorius*.
- CDK2 signals are distributed along the sex bivalent as well as or close to autosomal bivalents:
  5. Unusual female XX with a delayed synapsis, as in *E. tancrei*.

The CDK2 localization analysis in all types of sex bivalents allowed us to determine general patterns (it was summarized in Figure 6 and Table S1):

- Telomeric ends are CDK2-positive;
- If a recombination nodule is formed, then CDK2 is localized in it;
- The X chromosome, as a rule, has a large or clearly visible CDK2 signals in the asynaptic region;
- The short Y chromosome is deficient in CDK2 (single faint signals or none at all).

We observed some trends in CDK2 manifestation in meiotic sex chromosomes. First, we detected a variable CDK2 pattern in the asynaptic regions of the sex bivalents (rat, mole rat, vole XY, mole vole male XX vs. hamster XY). The presence of numerous CDK2 signals on the X and a rare, weak CDK2 signal on the Y, particularly in the early stages of prophase I of meiosis, is subject to further study. Indeed, the role of CDK2 kinase in the unsynapsed axial elements of sex bivalents is still not known. It has been hypothesized that CDK2 interacting with other proteins may be part of a more tightly regulated checkpoint control [51,52]. It is possible that the interaction of CDK2 with participants of the MSCI process may be involved in germ cells progression through the system of meiotic checkpoints. This hypothesis requires further testing. Extensive study of the relationship of CDK2 with MSCI markers such as ATR, BRCA1, and others may help in deciphering its role in the asynaptic segments of prophase I sex chromosomes.

Second, CDK2 distribution did not depend on the synapsis and recombination of sex chromosomes (synaptic chiasmatic rat, mole rat XY, mole vole male XX vs. asynaptic achiasmatic vole XY). CDK2 manifestation in the asynaptic and achiasmatic XY of voles and non-standard male XX of mole voles resembled the classical XY pattern of mouse, rat, and mole rat.

Third, we revealed a conservative pattern in CDK2 distribution at the interstitial sites and telomeric ends.

Thus, we observe a divergent evolution in relation to CDK2 distribution in the sex chromosomes of different rodent groups: On the one hand, some variability, on the other hand, high conservation. Probably, this variability may be due to possible transformations in the sex chromosome structure, such as the same size X and Y in hamsters. These assumptions can be confirmed in other studies.

## 4. Material and Methods

### 4.1. Animals

We studied two males of the rat *Rattus norvegicus,* line Dark Agouti, three males of the mole vole *E. talpinus*, two females of the mole vole *Ellobius tancrei,* one male of the mole vole *Ellobius alaicus*, one male of the vole *Microtus arvalis*, one male of the hamsters *Cricetulus migratorius,* and two males of the Middle East blind mole rat *Nannospalax leucodon*. Manipulations with animals were carried out according to the international rules [53] and the rules of the Ethical Committee of Vavilov Institute of General Genetics RAS (order No. 3 of 10 November 2016).

### 4.2. Meiotic Samples Preparing and Its Analyses

Synaptonemal complex (SC) preparations were made and fixed using the technique described previously [40] or using the technique of Peters et al. [54] with modifications [55]. The statistical analysis of all data was performed using GraphPad Prism 9 software (San Diego, CA, USA). Mean values (M) and the standard error of the mean (SEM) were calculated by the descriptive option of the software (Figure S5, Tables S2, S3). *p*-values reported in Table S2 were calculated by Mann–Whitney two-sided non-parametric test.

To assess colocalization/non-colocalization of the MLH1/CDK2 and SUN1/CDK2 pairs, 30 and 20 pachytene spermatocytes were analyzed, respectively. If the same bivalent had MLH1 (or SUN1) and CDK2 signals at one point, then this was interpreted as colocalization. All data are summarized in (Tables S3 and S4).

### 4.3. Antibodies

Antibodies, used for immunostaining: rabbit anti-SYCP3 antibodies (diluted 1:250, Abcam, Cambridge, UK) as a marker for lateral elements of SC and axial elements; human anti-centromere antibody CREST (1:250, Fitzgerald Industries International, Concord, MA, USA) for detecting kinetochores. Localization of CDK2 was detected applying mouse antibodies CDK2 (1:250, Santa Cruz Biotechnology Inc., Santa Cruz, CA, USA). DNA double-strand break (DSB) loci were immunostained with mouse antibodies against the RAD51 protein (1:200, Abcam, Cambridge, UK). Late recombination nodules were detected using mouse antibodies MLH1 (1:50, Abcam, Cambridge, UK). Proteins involved in the telomere attachment to nuclear envelope SUN1 were distinguished using rabbit antibodies SUN1 (1:250, Abcam, Cambridge, UK). As secondary antibodies we used goat anti-rabbit IgG, Alexa Fluor 488-conjugate (Invitrogen, Carlsbad, CA, USA); goat anti-human IgG, Alexa Fluor 546-conjugate (Invitrogen, Carlsbad, CA, USA); goat anti-mouse IgG, Alexa Fluor 546-conjugate and IgG, Alexa Fluor 555-conjugate (Invitrogen, Carlsbad, CA, USA) (diluted 1:300–800). Slides were washed in phosphate-buffered saline (PBS) and immersed into Vectashield with 4′,6-diamidino-2-phenylindole (DAPI) (Vector Laboratories, Burlingame, CA, USA). Slides were analyzed using a fluorescence light microscope Axio Imager D1 (Carl Zeiss, Jena, Germany).

### 4.4. Immunostaining Procedure

Immunostaining was performed using our protocol of multi-round staining. The scheme of the procedures is shown in Figure S4.

In the first round, pairs of antibodies SYCP3/CDK2, SUN1/CDK2, and SYCP3/RAD51 or SYCP3/MLH1 were kept at +4 °C overnight (Figure S4, Step 1). After the incubation, slides were washed three times for 2 min in PBS, then the corresponding secondary antibodies were placed and kept for 4–6 h (Figure S4, Step 2). After a PBS wash and processing with Vectashield with DAPI, slides were examined using the fluorescence light microscope. 

The cells were photographed; their position was recognized using the coordinate grid of the microscope and recorded in the work log. Then the slides were washed 4–6 times in PBS for 5–6 min. The second round included staining with the next primary antibodies (Step 3), washing in PBS and incubation with appropriate secondary antibodies (Step 4). Again, the cells were examined under a microscope and photographed, and then the slides were washed carefully as in previous round. During the third round, the third set of primary antibodies was used (Step 5), then slides were washed in PBS and incubated with the third set of secondary antibodies (Step 6), followed by photography.

Thus, we observed the distribution of four antibodies in the same cells. We have the experience of immunostaining by a larger number of rounds [25]. It is important to note that a satisfactory result of multi-round immunostaining can be obtained, taking into account several important rules:

1. The fluorescence of the dyes conjugated to the secondary antibody should be burned between rounds by keeping the slides under the halogen lamp and thorough washing in PBS. The duration of the washing between rounds is always longer than the washings within procedures of each round.
2. It is necessary to consider the size of the intra-nuclear structure, the fluorescence intensity of each fluorochrome and the features of its burnout under a halogen lamp. In each case, the volume of primary and secondary antibodies, as well as the concentration of antibodies applied to the slides should be under controlled. For example, secondary antibodies Alexa Fluor 555 were more resistant to burnout, so we used them in the second or third rounds.
3. In the first round, more miniature structures should be immuno-stained because such structures tend to have a less intense glow. The order of applying antibodies should be chosen due to the researcher’s experience of examining the staining slides. The illumination of the fluorochromes associated with small structures, as a rule, was eliminated faster and easier when burning under the halogen lamp. However, for example, CREST antibodies in many species demonstrated bright signals, the glow of which was able to obscure (overlay) the light of other signals in the next rounds, so we used these antibodies in the last steps of immunostaining.
4. Multi-round immunostaining should be accompanied by a specific single-round one. The results of both approaches should be used to correctly assess distribution of immuno-signals in cells.

## 5. Conclusions

Immunocytochemical analysis of CDK2 showed variable patterns in the asynaptic regions of heteromorphic sex chromosomes and conserved ones at the interstitial regions and telomeric ends. The variations in CDK2 distribution are presumably associated with differences in the sex chromosome system: from isomorphic sex chromosomes (mole voles) through intermediate stages (classical heteromorphic sex chromosomes with a small synaptic zone in the mole rat, rat, and hamster) to full asynapsis of heteromorphic sex chromosomes, which was caused, apparently, by the loss of a PAR in the common vole. Manifestation the CDK2 were detected even when sex chromosomes are asynaptic, possibly because of other function or insufficient suppression of the kinase activity in a case of sex chromosomes rapid evolution. In the homomorphic XX sex chromosomes, CDK2 signals was revealed as single foci in one synaptic segment. Localization of CDK2 signals at the telomeric regions of synaptic area of sex chromosomes was revealed for all studied species. This tendency corresponds to that previously shown for mice. 

Our studies revealed both a conservative manifestation pattern of this kinase and variability in the signal distribution depending on the heteromorphization of the sex chromosomes. These data highlight the importance of continuing to study the role of the kinase in meiosis.

## Data Availability

Not applicable.

## Supplementary Materials

Supplementary materials can be found at https://www.mdpi.com/1422-0067/22/4/1969/s1. Table S1. Sex chromosomes of rodents studied and CDK2 signals patterns. Figure S1. CDK2 distributions in prophase I sex chromosomes of different rodent taxa. Figure S2. CDK2 distributions in pachytene meiocytes of different rodents. Figure S3. CDK2 distributions in prophase I spermatocytes of *Microtus arvalis*. Figure S4. Multi round/multistep sequential immunostaining procedure. Table S2. Rodent sex bivalents and CDK2 foci in spermatocytes at the early - mid pachytene stage. Figure S5. A number of CDK2 foci (M ± SEM) per asynaptic part of the sex chromosome in pachytene meiocytes of different rodents. Table S3. Colocalization and non-colocalization of SUN1 and CDK2 foci in autosomal bivalents and sex bivalents of rat and mole vole spermatocytes at the mid pachytene stage. Table S4. Colocalization and non-colocalization of MLH1 and CDK2 foci in autosomal bivalents of rat and mole vole spermatocytes at the mid pachytene stage.

## Abbreviations

CDK2	Cyclin-dependent kinases 2
PAR	Pseudoautosomal region
SC	Synaptonemal complex
SYCP3	Synaptonemal complex protein 3
DSB	Double-strand break
CREST	Calcinosis Raynaud’s phenomenon, Esophageal dysmotility, Sclerodactyly, and Telangiectasia
MLH1	MutL homolog 1
BRCA1	Breast cancer 1
MSCI	Meiotic sex chromosome inactivation
SUN1	Sad1 and UNC84 domain-containing protein 1
PBS	Phosphate buffered saline
RPA	Replication protein A
