# Supplementary material for: Kinase CDK2 in Mammalian Meiotic Prophase I: Screening for Hetero- and Homomorphic Sex Chromosomes
Source: Int J Mol Sci. 2021 Feb 17;22(4):1969. doi: 10.3390/ijms22041969 (PMC7922030; doi:10.3390/ijms22041969)
Supplement: Supplementary file 1 [file ijms-22-01969-s001.pdf]

International Journal of Molecular Sciences

MDPI

Supplemental materials for:

Kinase CDK2 in mammalian meiotic prophase I: screening for hetero- and homomorphic sex chromosomes

Sergey Matveevsky[1,*], Tsenka Chassovnikarova[2,3], Tatiana Grishaeva[1], Maret Atsaeva[4], Vasilii Malygin[5], Irina Bakloushinskaya[6,†] and Oxana Kolomiets[1,†]

[1] Laboratory of Cytogenetics, Vavilov Institute of General Genetics, Russian Academy of Sciences, 119991 Moscow, Russia; grishaeva@vigg.ru (T.G.), olkolomiets@mail.ru (O.K.)

[2] Department of Animal Diversity and Resources, Institute of Biodiversity and Ecosystem Research, Bulgarian Academy of Science, 1000 Sofia, Bulgaria; t.tchasovnikarova@gmail.com (T.C.)

[3] Department of Zoology, Biological Faculty, University "Paisi Hilendarski", 4000 Plovdiv, Bulgaria

[4] Department of Cell Biology, Morphology and Microbiology, Chehen State University, 364051 Grozny, Chechen Republic, Russia; acaeva-mm@mail.ru (M.A.)

[5] Department of Vertebrate Zoology, Lomonosov Moscow State University, Biological Faculty, 119991 Moscow, Russia; vmalygin@mail.ru (V.M.)

[6] Laboratory of Genome Evolution and Speciation, Koltzov Institute of Developmental Biology, Russian Academy of Sciences, 119334 Moscow, Russia; irina.bakl@gmail.com (I.B.)

* Correspondence: sergey8585@mail.ru

† These authors contributed equally

Table S1. Sex chromosomes of rodents studied and CDK2 signals patterns

Species	Chromosome numbers	Sex chromosomes*	Sex bivalent*	CDK2*	Refs
Rattus norvegicus	2n=42, NF=60, ♂XY,♀XX	Heteromorphic system. X: medium acrocentric Y: short acrocentric	Synaptic XY bivalent. The centromeric region of the X participates in the synapsis. The Y axis lies completely along the X in the mid-late pachytene	X and Y have CDK2 positive MLH1 containing recombination nodules and CDK2 positive telomeres. Asynaptic part of the X has a large number of CDK2 signals. Y has on average 1 weak CDK2 signal or it is completely absent.	[1,2]
Ellobius talpinus	2n=54, NF=54, ♂♀XX	Male and female large homomorphic / isomorphic (G-bands) acrocentric sex chromosomes	Synaptic male XX bivalent with two telomeric SC segments and extended central zone of asynapsis	X and X have CDK2 positive MLH1 containing recombination nodules of the synaptic segments and CDK2 positive telomeres. Asynaptic part of the X has a large number of CDK2 signals.	[3,4]
Ellobius alaicus	2n=52-48, NF=56, ♂♀XX	Male and female large homomorphic / isomorphic (G-bands) acrocentric sex chromosomes	Synaptic male XX bivalent with two telomeric SC segments and extended central zone of asynapsis	X and X have CDK2 positive telomeres and regularly, but not always one CDK2 signal in the synaptic segments. Asynaptic part of the X has a large number of CDK2 signals.	[4,5]
Ellobius tancrei	2n=54-30, NF=56, ♂♀XX	Male and female large homomorphic / isomorphic (G-bands) acrocentric sex chromosomes	Synaptic female XX bivalent with delayed synapsis in the central area	Female XX bivalent has CDK2 positive telomeres and 2-4 weak CDK2 signals in interstitial sites and delayed area.	[3,4]
Microtus arvalis	2n=46, NF=84, ♂XY,♀XX	Heteromorphic system. X: medium metacentric Y: short acrocentric	Asynaptic achiasmatic XY bivalent. There are irregular SYCP3 thickenings along the X and Y axes. The X and Y axes are always close to each other	X and Y have CDK2 positive telomeres. X has a large number of CDK2 signals. Y has on average 1 weak CDK2 signal or it is completely absent.	[6,7]
Cricetulus migratorius	2n=22, NF=44, ♂XY,♀XX	Heteromorphic system. X: large submetacentric Y: large submetacentric X and Y chromosomes are the same length and different G-band patterns	Synaptic XY bivalent with long synaptic segments and thick long asynaptic axes that have numerous SYCP3 loops and SYCP3 fragments	X and Y have CDK2 positive telomeres and one CDK2 signal in synaptic segment. Asynaptic parts and SYCP3 loops/fragments of the X and Y have 4-8 CDK2 dots of different intensity and size.	[8,9]
Nannospalax leucodon	2n=46-58, NF=76-98, ♂XY,♀XX	Heteromorphic system. X: medium submetacentric Y: short subtelocentric	Synaptic XY bivalent with centromere coorientation. The Y axis lies completely along the X in the mid-late pachytene	X and Y have CDK2 positive telomeres. Asynaptic part of the X has a large number of CDK2 signals. Y has on average 1 weak CDK2 signal or it is completely absent.	[10]

Remarks: 2n – diploid number, NF – number of chromosome arms, SC – synaptonemal complex. * - see Figure S1

International Journal of Molecular Sciences

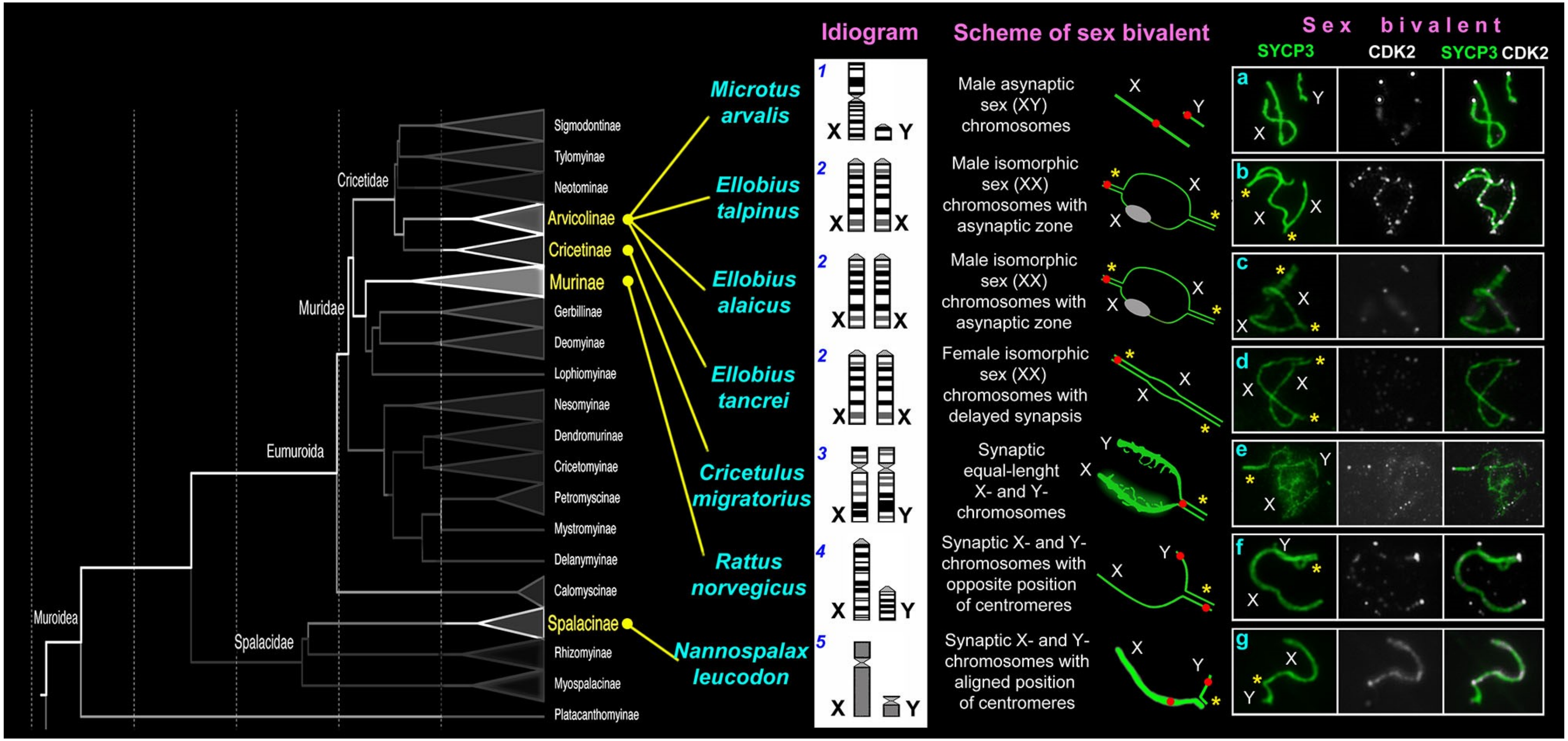

Figure S1. CDK2 distributions in prophase I sex chromosomes of different rodent taxa. The phylogenetic tree is taken from Steppan, Schenk, 2017 [11], doi: 10.1371/journal.pone.0183070.g001 (with modifications: resized, desaturated, recolored and deleted some taxa names) in accordance the Creative Commons Attribution License (CC BY 4.0). Idiograms of G-banded sex chromosomes were drawn based on: 1. Mazurok et al., 1996 [6]; 2. Matveevsky et al., 2017 [4]; 3. Lavappa, 1977 [8]; 4. Hamta et al., 2006 [2]; 5. Savić, I., Soldatović, 1984 [10] (G-banding is unknown). Each sex bivalent of the rodent can be seen in the whole nucleus: **a** in Figure S2a, **b** in Figure 4d, **c** in Figure S2b, **d** in Figure S2c, **e** in Figure S2d, **f** in Figure S2e, **g** in Figure S2f. A yellow asterisk indicates the terminal synapsis of the sex chromosomes.

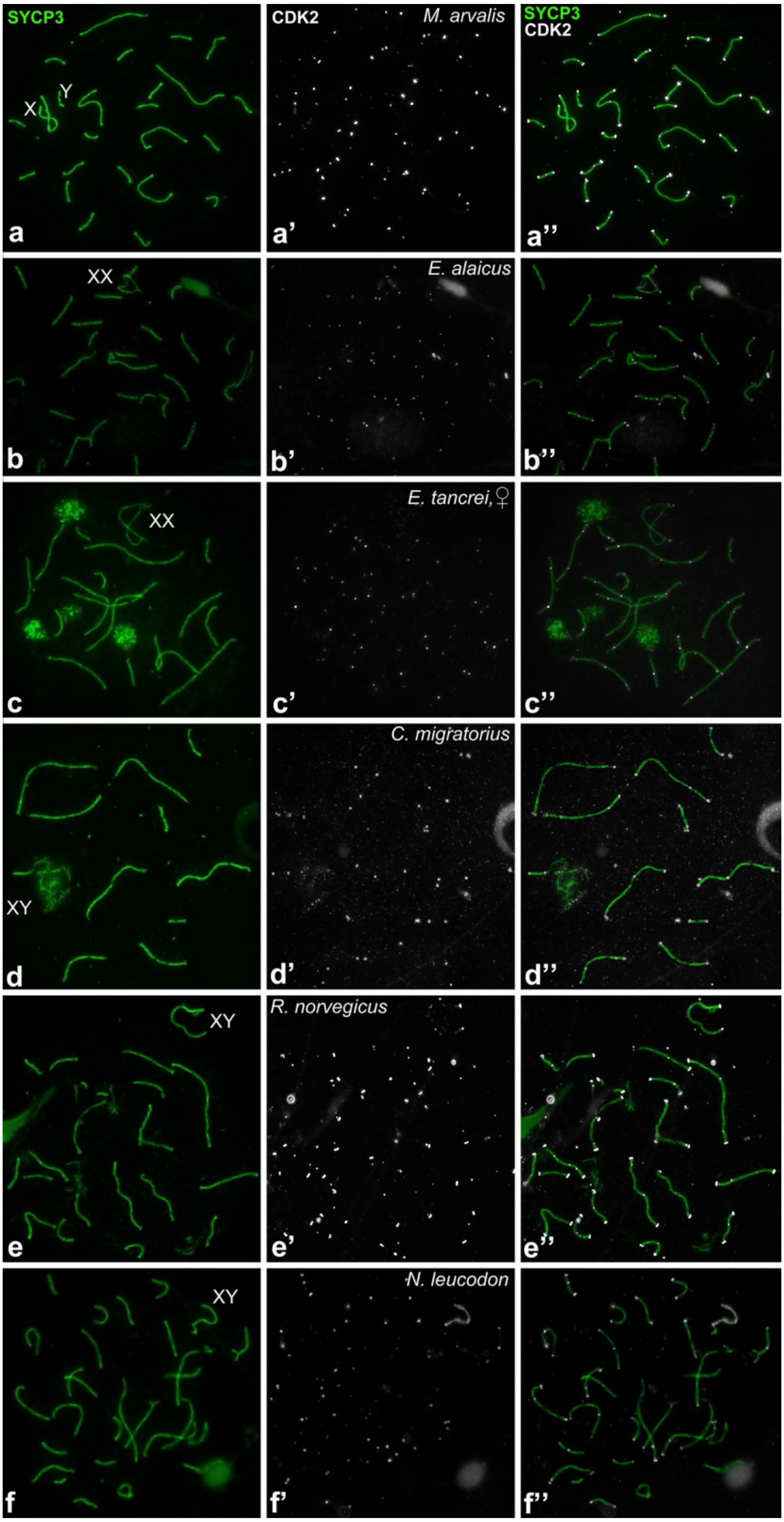

Figure S2. CDK2 distributions in pachytene meiocytes of different rodents. **a-a''.** *Microtus arvalis*; **b-b''.** *Ellobius alaicus*; **c-c''.** *Ellobius tancrei*; **d-d''.** *Cricetulus migratorius*; **e-e''.** *Rattus norvegicus*; **f-f''.** *Nannospalax leucodon*. a-b, d-f – males; c – female.

International Journal of
Molecular Sciences

MDPI

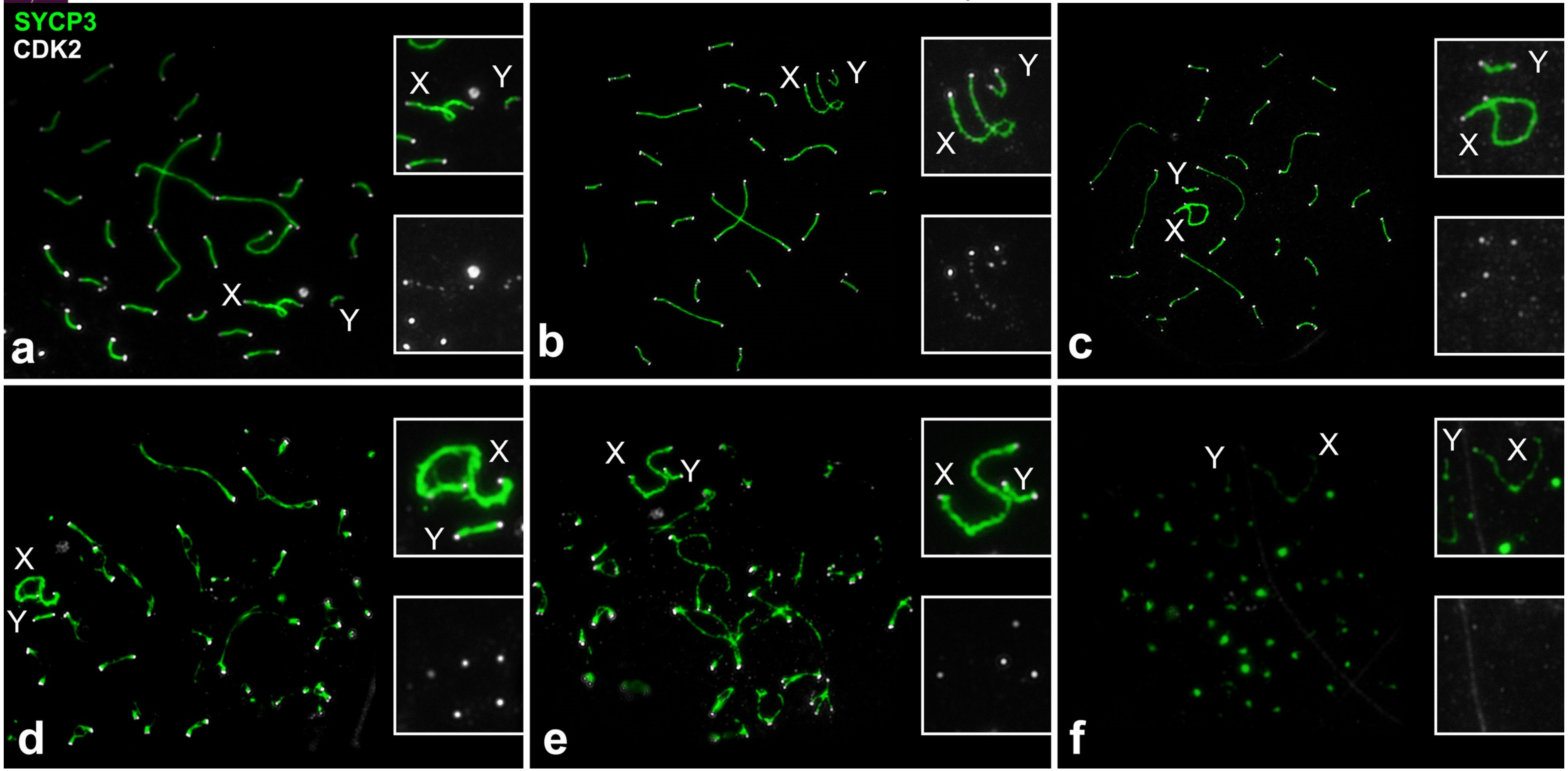

Figure S3. CDK2 distributions in prophase I spermatocytes of *Microtus arvalis.* **a.** Early pachytene; **b.** Mid pachytene; **c.** Mid – Late pachytene; **d.** Early diplotene; **e.** Mid diplotene; **f.** Late diplotene - Diakinesis.

International Journal of
Molecular Sciences

MDPI

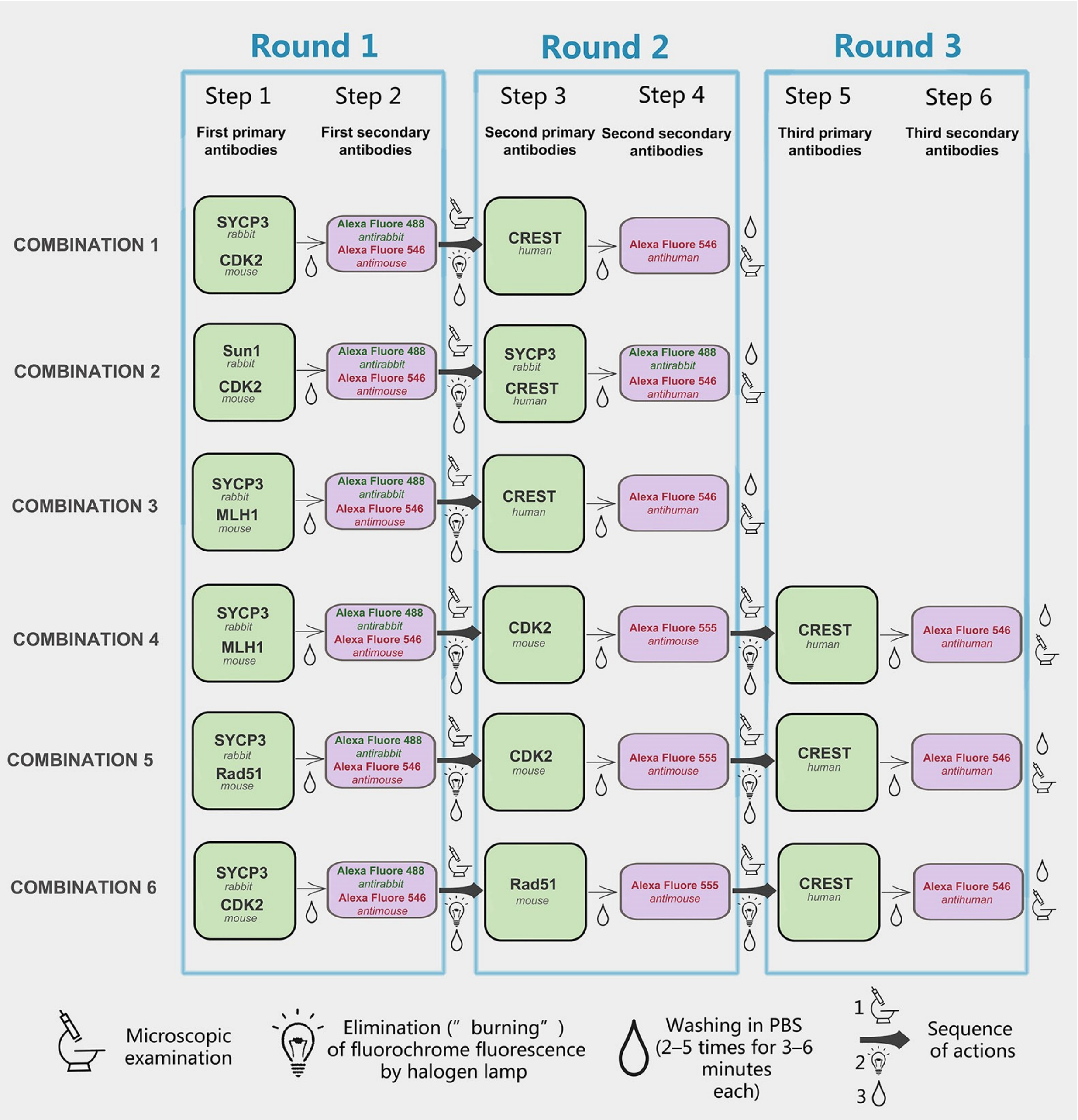

Figure S4. Multi round/multistep sequential immunostaining procedure. Immunostaining was carried out sequentially in several rounds and steps. See detailed description in "Immunostaining procedure" subsection (Materials and Methods section).

International Journal of Molecular Sciences

MDPI

Table S2. Rodent sex bivalents and CDK2 foci in spermatocytes at the early - mid pachytene stage

	Species	Number of nuclei analyzed#	Average length of the X, µm [M±SEM]	Average number of CDK2 foci in the X [M±SEM] (see Figure S5)	Number of CDK2 foci per length of the X	Average length of the Y, µm [M ±SEM]	Average number of CDK2 foci in the Y [M ±SEM]	Number of CDK2 foci per length of the Y
	1	*2*	*3*	*4*	*5*	*6*	*7*	*8*
A	*Rattus norvegicus*, ♂	31	12.8±0.8	15.8±0.6, range: 10-22	1.23	6.49±0.37	1.42±0.17, range: 0-4	0.22
B	*Ellobius talpinus*, ♂	38	8.2±0.6	9.83±0.49, range: 6-18	1.19	10.05±0.7	X*: 8.08±0.39, range: 3-14	0.8
C	*Ellobius alaicus*, ♂	16	8.15±0.76	7.87±0.67, range: 4-13	0.97	9.84±0.63	X*: 6.63±0.64, range: 3-11	0.67
D	*Ellobius tancrei*, ♀	22	11.07±0.48	XX**: 3.63±0.36, range: 1-8	0.33	–	*See 4D*	–
E	*Nannospalax leucodon*, ♂	28	7.97±0.59	10.37±0.74, range: 6-19	1.3	3.31±0.26	1.2±0.16, range: 0-3	0.36
F	*Cricetulus migratorius*, ♂	21	16.41±0.73	5.76±0.44, range: 2-10	0.35	15.29±0.71	4.47±0.43, range: 1-9	0.29
G	*Microtus arvalis*, ♂	44	10.76±0.8	5.4±0.48, range: 1-16	0.5	3.58±0.29	1.06±0.13, range: 0-3	0.3

The nuclei with sex chromosomes were analyzed during the transition from early to mid pachytene stages when it is possible to identify individual axial elements in the sex bivalent. Sex bivalents with fragments of axial elements and with complete synapsis of the X and Y and CDK2 foci at the telomere sites were not taken into account. * The mole voles have two male X chromosomes; the calculations took into account the second X chromosome with a chromatin dense body (ChB). ** The whole sex (XX) bivalent with delayed synapsis was taken into account for *E. tancrei* female. M – mean value. SEM - the standard error of the mean.

Comparison of the number of CDK2 foci for some pairs:

Significant ($P < 0.05$): A4/B4, A4/B7, A4/C4, A4/C7, A4/D4, A4/E4, A4/F4, A4/G4, B4/B7; B4/C4, B4/C7, B4/D4, B4/E4, B4/F4, B4/G4, C4/D4, C4/E4, C4/F4, C4/G4, D4/E4/ D4/F4, D4/G4, E4/F4, E4/G4, B7/C7;

Not significant: C4/C7, F4/F7, F4/G4

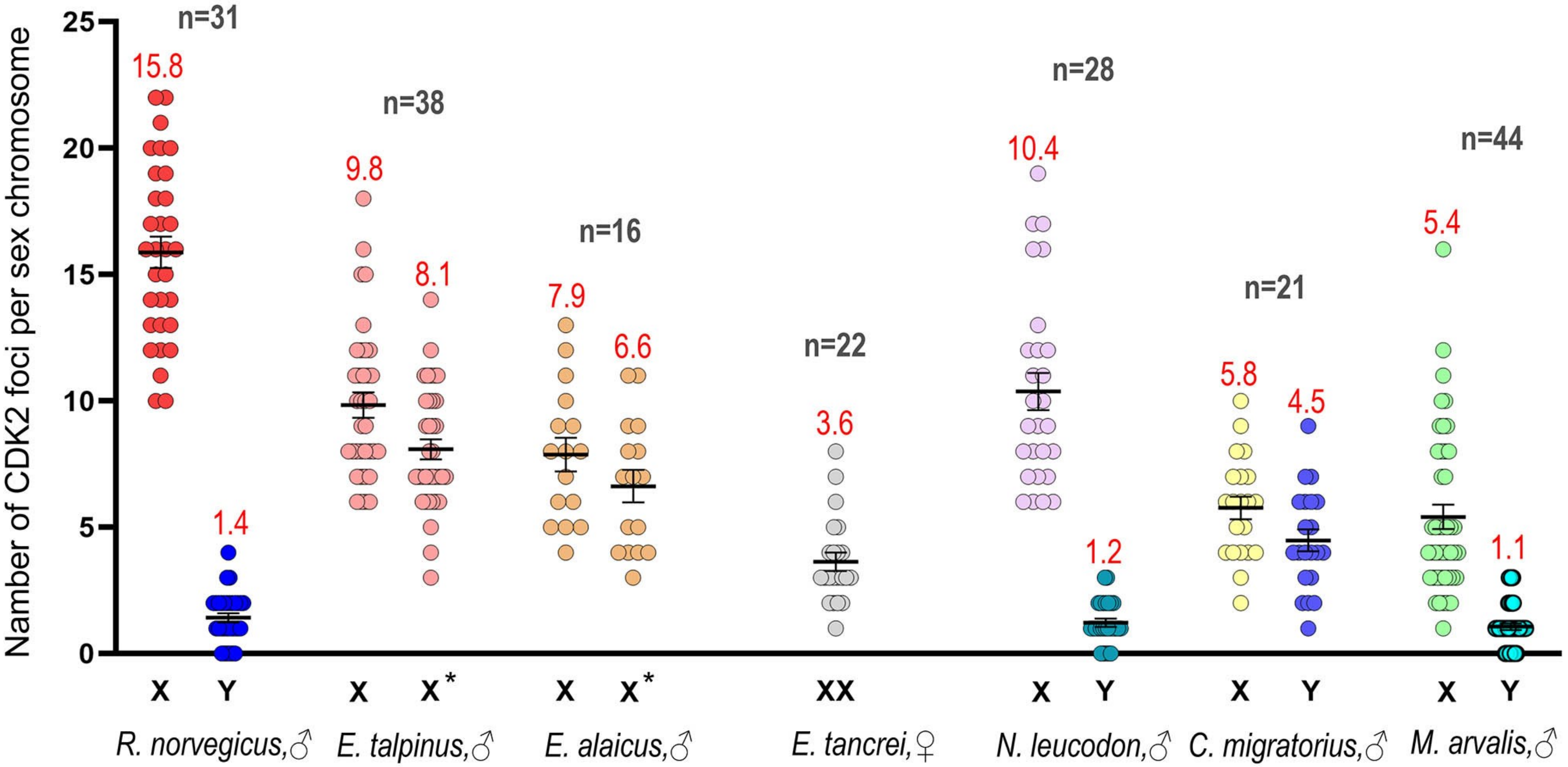

Figure S5. A number of CDK2 foci (M±SEM) per asynaptic part of the sex chromosome in pachytene meiocytes of different rodents. Sex chromosomes were analyzed during the transition from early to mid pachytene stages when it is possible to identify individual axial elements in the sex bivalent. The whole bivalent was taken into account for *E. tancrei* female. X* indicates male X with a chromatin-dense body for *Ellobius*. Data of CDK2 signals see Table S2. n – number of cells.

International Journal of *Molecular Sciences*

MDPI

Table S3. Colocalization and non-colocalization of SUN1 and CDK2 foci in autosomal bivalents and sex bivalents of rat and mole vole spermatocytes at the mid pachytene stage

Species	Number of nuclei	Total number of bivalents	Bivalents with colocalization of SUN1 and CDK2	Bivalents with non-colocalization of SUN1 and CDK2 [n (%), orange color]	Average number of bivalents with non-colocalization per nucleus [n±SEM]
Rattus norvegicus	20	420	408	12 (2,9%) 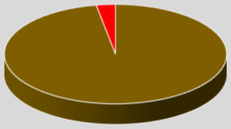	0.6±0.19 (range: 0-4)
Ellobius talpinus	20	540	533	7 (1,3%) 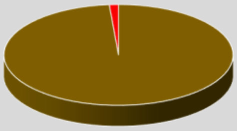	0.35±0.12 (range: 0-2)

Remarks to diagrams: brown – colocalization, red – non-colocalization

Table S4. Colocalization and non-colocalization of MLH1 and CDK2 foci in autosomal bivalents of rat and mole vole spermatocytes at the mid pachytene stage

Species	Number of nuclei	Total number of autosomal bivalents	Autosomal bivalents with colocalization of MLH1 and CDK2	Autosomal bivalents with non-colocalization of MLH1 and CDK2 [n (%), orange color]	Average number of bivalents with non-colocalization per nucleus [n±SEM]
Rattus norvegicus	30	600	567	33 (5.5%) 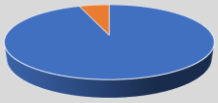	1.1±0.23 (range: 0-5)
Ellobius talpinus	30	780	762	18 (2.3%) 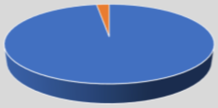	0.6±0.15 (range: 0-3)

Remarks to diagrams: blue – colocalization, orange – non-colocalization

References

1. Ricciuti, F.; Ruddle, F.H.; Cox, D.M.; Bobrow, M.; Madan, K.; Pathak, S., ... & Chen, T. R. (1973). Standard karyotype of the Norway rat, *Rattus norvegicus*. *Cytogenetic and Genome Research*, **1973**. *12*(3), 199-205.
2. Hamta, A.; Adamovic, T.; Samuelson, E.; Helou, K.; Behboudi, A.; Levan, G. Chromosome ideograms of the laboratory rat (Rattus norvegicus) based on high-resolution banding, and anchoring of the cytogenetic map to the DNA sequence by FISH in sample chromosomes. *Cytogenetic and genome research*, **2006**. *115*(2), 158-168. doi: 10.1159/000095237
3. Bakloushinskaya, I.Y.; Matveevsky, S.N.; Romanenko, S.A.; Serdukova, N.A.; Kolomiets, O.L.; Spangenberg, V.E.; Lyapunova, E.A.; Graphodatsky, A.S. A comparative analysis of the mole vole sibling species *Ellobius tancrei* and *E. talpinus* (Cricetidae, Rodentia) through chromosome painting and examination of synaptonemal complex structures in hybrids. *Cytogenet. Genome Res.* **2012**, *136*, 199–207. doi: 10.1159/000336459
4. Matveevsky, S.; Kolomiets, O.; Bogdanov, A.; Hakhverdyan, M.; Bakloushinskaya, I. Chromosomal evolution in mole voles *Ellobius* (Cricetidae, Rodentia): Bizarre sex chromosomes, variable autosomes and meiosis. *Genes* **2017**, *8*, 306. doi: 10.3390/ijms21207630
5. Bakloushinskaya, I.; Lyapunova, E.A.; Saidov, A.S.; Romanenko, S.A.; O'Brien, P.C.; Serdyukova, N.A.; Ferguson-Smith, M.; Matveevsky, S.N.; Bogdanov, A. Rapid chromosomal evolution in enigmatic mammal with XX in both sexes, the Alay mole vole *Ellobius alaicus* Vorontsov et al., 1969 (Mammalia, Rodentia). *Comp. Cytogenet.* **2019**, *13*, 147–177. doi: 10.3897/CompCytogen.v13i2.34224

6. Mazurok, N.A.; Isaenko, A.B.A.; Nesterova, T.B.; Zakian, S.M. High-resolution G-banding of chromosones in the Common vole *Microtus arvalis* (Rodentia, Arvicolidae). *Hereditas*, **1996**. *124*(3), 229-232. doi: 10.1111/j.1601-5223.1996.00229.x

7. Ashley, T.; Jaarola, M.; Fredga, K. Absence of synapsis during pachynema of the normal sized sex chromosomes of *Microtus arvalis*. *Hereditas*, **1989**. *111*(3), 295-304. doi: 10.1111/j.1601-5223.1990.tb00408.x

8. Akhverdian, M.R. Specialities of behaviour of the sex chromosomes in meiosis in the Armenian hamster (*Cricetulus migratorius* Pallas, 1770). Genetica (Rus.). **1993**. *29*: 950–959.

9. Lavappa, K.S. Chromosome banding patterns and idiogram of the Armenian hamster, *Cricetulus migratorius*. *Cytologia*, **1977**. *42*(1), 65-72. doi: 10.1508/cytologia.42.65

10. Savić, I.; Soldatović, B. Karyotype evolution and taxonomy of the genus *Nannospalax* Palmer, 1903, Mammalia, in Europe. *Serbian Acad. Sci. Arts Sep. Ed.* **1984**, *59*, 1–104.

11. Steppan, S.J.; Schenk, J.J. Muroid rodent phylogenetics: 900-species tree reveals increasing diversification rates. *PloS One*, **2017**. *12*(8), e0183070. doi: 10.1371/journal.pone.0183070
